# Endothelial MHC expression is required to initiate T cell–mediated rejection of 3D-printed skin grafts

**DOI:** 10.1172/jci.insight.201946

**Published:** 2026-04-22

**Authors:** Zuzana Tobiasova, Esen Sefik, Lingfeng Qin, Jennifer M. McNiff, Gwendolyn Davis, Richard A. Flavell, W. Mark Saltzman, Jordan S. Pober

**Affiliations:** 1 Department of Immunobiology,; 2 Department of Surgery,; 3 Department of Dermatology, and; 4 Department of Pathology Yale School of Medicine, Yale University, New Haven, Connecticut, USA.; 5 Howard Hughes Medical Institute, New Haven, Connecticut, USA.; 6 Department of Biomedical Engineering,; 7 Department of Chemical & Environmental Engineering, and; 8 Department of Cellular & Molecular Physiology, Yale University, New Haven, Connecticut, USA.

**Keywords:** Immunology, Vascular biology, Skin, Tolerance, Transplantation

## Abstract

Vascularized skins were 3D printed using single donor human fibroblasts, pericytes, keratinocytes, and endothelial cells (ECs), the latter either unmodified (WT-ECs) or deleted of MHC molecules (KO-ECs). Adult MISTRG6 immunodeficient mice neonatally inoculated with adult human hematopoietic stem cells (HSCs) received printed skin allogeneic to the HSCs and were boosted 3 weeks after grafting with human PBMCs autologous to the HSCs. HSC inoculation alone produced low levels of circulating human myeloid and lymphoid cells without affecting grafts; PBMC boosting dramatically increased circulating human CD4^+^ T cells and boosted CD8^+^ T cells only in mice with WT-EC grafts. These grafts became infiltrated by human macrophages, dendritic cells, CD4^+^ and CD8^+^ T cells and showed evidence of rejection. Shared T cell clones were present in skin and spleen. KO-EC grafts had minimal infiltration of graft or spleen without rejection, despite MHC molecule expression on other graft cell types.

## Abstract 2

<span>Allogeneic 3D-printed vascularized human skin transplanted to mice with human immune cells induced rejection. When vessel endothelial cells were modified, no transplantation rejection occurred.</span>

## Introduction

Tissue engineering can provide a potentially unlimited source of organs for therapeutic transplantation, but these tissue-engineered organs are currently restricted to thin constructs because they lack vascularization and must survive by nutrient and gas diffusion (1). Skin grafting is used to treat nonhealing ulcers and extensive skin tumor resections. Autologous skin transplantation is limited because the underlying disorders that lead to nonhealing ulcers, such as diabetes or advanced age, generally impair wound repair, complicating skin harvest (2). Allogeneic skin is among the tissues most prone to rejection (3). This need has driven development of synthetic skin substitutes. Burke and Yannas described clinical results using bilayered artificial skin as early as 1981 (4), and there are more than 10 different, approved synthetic skin substitutes that are either acellular or cellularized to form dermal, epidermal, or epidermodermal mimetics (5, 6). None are completely satisfactory (7).

Cellularized grafts respond better than acellular counterparts to environmental stresses and injuries, although acellular grafts may become cellularized after implantation through invasion of the graft material by recipient cells. To be clinically useful, grafts must be available “off the shelf.” Consequently, the currently available cellularized skin substitutes contain keratinocytes and/or fibroblasts that are allogeneic to the host. Surprisingly, unlike allogeneic natural skin, allogeneic cells in these synthetic skins do not initiate a recipient immune-mediated rejection response (8, 9). Nevertheless, the graft cells die and grafts often slough owing to the absence of microvascular graft perfusion (10). Incorporating a vascular network into the graft could avoid ischemic sloughing. A critical component of the microvascular circulation is the endothelial cell (EC) lining, which regulates blood flow, controls permselectivity, and minimizes thrombosis and inflammation (11). Moreover, cultured human ECs can spontaneously organize into a tubular network capable of perfusing acellular collagen gels or decellularized human skin (12, 13), suggesting that inclusion of ECs in a synthetic graft could provide adequate perfusion in skin and perhaps other tissues (14).

The absence of immune rejection of constructs containing allogeneic cells is puzzling but can be explained in two ways: either grafts lack specific cell types capable of alloantigen presentation to resting T cells, such as myeloid or lymphoid cells resident within normal tissues, or, alternatively, absence of a vascular system within the graft limits contact between recipient alloreactive T cells and graft cells. Thus, including ECs in grafts to form blood vessels may address lack of perfusion; it may simultaneously lead to rejection regardless of which explanation is true because human ECs express and can present both class I and II MHC molecules, activating both alloreactive CD4^+^ and CD8^+^ effector memory T cells (15). Alloreactive effector memory T cells arise as a result of prior infections because some T cell receptors (TCRs) for antigen-specific pathogen peptide that bound to self MHC cross react to non-self MHC molecules independent of the bound peptide (16). In clinical kidney transplantation, rejection is best predicted by the frequency of preexisting alloreactive effector memory T cells rather than total alloreactive T cells (17), and in immunodeficient mice bearing human skin grafts, adoptively transferred human effector memory T cells are the only T cell subpopulation capable of causing acute cell-mediated rejection (18). Much of the acute rejection response is mediated by CD8^+^ effector T cells, also known as cytotoxic T lymphocytes (CTLs), that directly recognize graft class I MHC molecules, but activated host CD4^+^ T cells can boost the CD8^+^ T cell response or activate natural killer cells or macrophages (19). Activation of host CD8^+^ or CD4^+^ T cells may involve recognition of non-self class I or II MHC molecules, respectively, on a graft-derived EC (called “direct recognition”) or on a host myeloid or lymphoid antigen-presenting cell that has acquired intact MHC molecules shed from the graft (called “semidirect recognition”). A third pathway (“indirect recognition”) involves host antigen-presenting cells acquiring peptides from allogeneic graft proteins and presenting them to host CD4^+^ T cells, but this process typically occurs with some delay. CRISPR-mediated gene disruption can be used to eliminate the expression of both class I and II MHC molecules on ECs, ablating their capacity to directly initiate an allogeneic response without compromising their capacity to self-assemble into blood vessels (20). Microvessels formed from such knockout ECs (KO-ECs) could still promote contact of T cells with graft cells but could no longer act as antigen-presenting cells.

**Figure 1:**
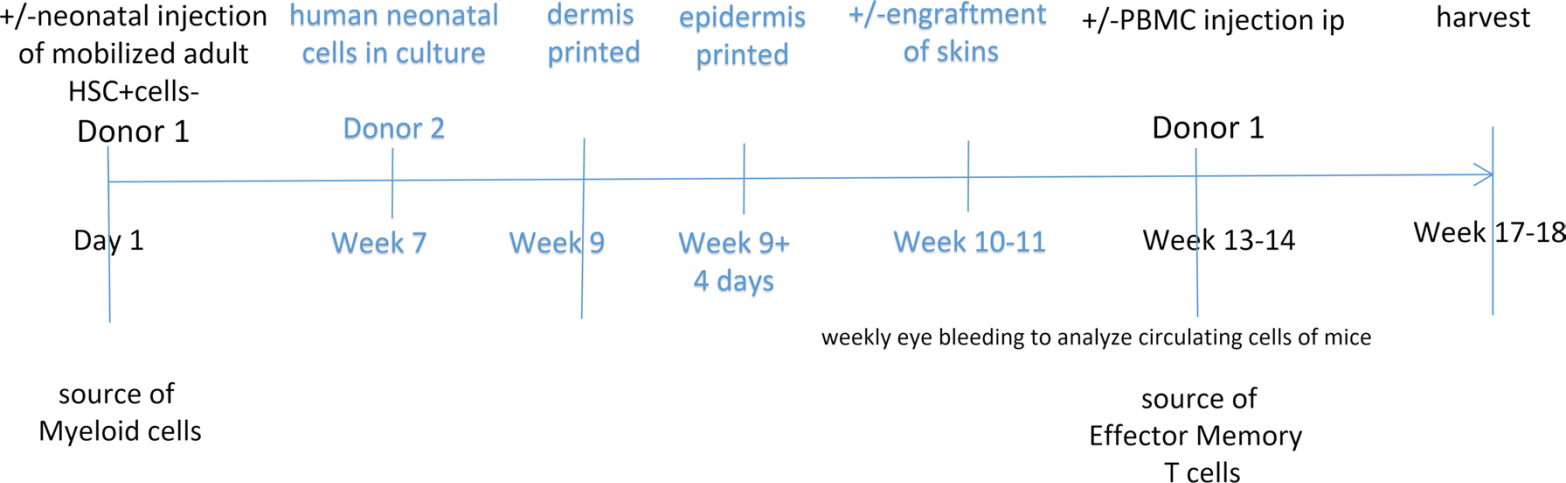
Experimental approach. Timelines of human HSC inoculation, 3D-printed skin grafting, and PBMC boosting using MISTRG6 mice.

We and others have studied the process of skin graft rejection using immunodeficient mouse strains in which the human immune system is partly reconstituted using hematopoietic cells from a donor allogeneic to the skin (12). The majority of human immune system mouse models, including the ones we have used in the past, are deficient in reconstitution of human myeloid cells (21) and may be of limited use in assessing rejection initiated by indirect or semidirect recognition. The MISTRG6 mouse — constructed by incorporating several human genes that support hematopoietic development into an immunodeficient BALB/c-derived strain lacking both Rag2 and the cytokine common **γ** chain (Il2rg^–/–^) — was developed to provide conditions that allow both human lymphoid and myeloid cells to be engrafted (22, 23). When these animals are injected as neonates with human hematopoietic stem cells (HSCs) isolated from human fetuses, neonates, or adults, they develop robust and functional circulating populations of myeloid monocyte/macrophages and dendritic cells in addition to T and B cells (22, 23). However, these animals are generally raised in specific pathogen–free environments and, thus, lack the history of infections required to generate alloreactive T memory populations, the principal orchestrators of acute cell-mediated allograft rejection (24). For this study, we developed an approach for using the MISTRG6 mouse strain in which neonates were inoculated with mobilized adult human HSCs and then engrafted with 3D-printed vascularized human skin grafts formed with a single donor source of keratinocytes, dermal fibroblasts, pericytes, and either MHC-expressing ECs (WT-ECs) or MHC-deleted ECs (KO-ECs). Three weeks later, the animals were boosted by injection of PBMCs from the same adult donor as the HSCs, thereby engrafting alloreactive effector memory T cells in an animal with functional human myeloid antigen-presenting cells (Figure 1). We used this model to study the human allogenic immune response to 3D-printed human skin grafts made with either unmodified (WT) ECs or ECs in which both class I and class II MHC molecules have been deleted, allowing us to separate the effects of increased vascularization from the immunological role of ECs.

## Results

### Hematopoietic reconstitution of skin-engrafted mice and 3D-bioprinted skin grafts in vivo.

The goal of our study was to determine the potential consequences of vascularizing bioengineered human skin using self-assembly of human ECs to form microvessels, with pericytes and fibroblasts provided as supportive elements. As described in the Methods, all cells in the skin construct were from the same donor, and the ECs were either unmodified (WT-ECs) or altered using CRISPR/Cas9 to ablate expression of both class I and class II HLA molecules (KO-ECs). This allowed us to separate the roles of microvascular ECs as a source of perfusion and as a potential initiator of graft rejection. To create our model, we introduced several elements into use in human immune system mice to study transplantation. Although MISTRG6 mice inoculated as neonates with human HSCs develop functional human myeloid as well as lymphoid cells (25), these animals do not develop human alloreactive memory T cells when raised in specific pathogen–free animal care facilities. To address this, we combined neonatal inoculation with human HSCs and boosting of the same animals as adults with PBMCs, a source of alloreactive memory, from the same donor. Each new component was tested in preliminary experiments described in the Methods. Our final experimental scheme is summarized in Figure 1. Mice were sacrificed approximately 6–7 weeks after skin engraftment, at which time blood, grafts, and spleen were examined by histology and flow cytometry. We did not see substantial variation among litters or between sex of the mice or between results with different skin cell donors (all male, of necessity since foreskin supplies two of the cell types utilized).

**Figure 2:**
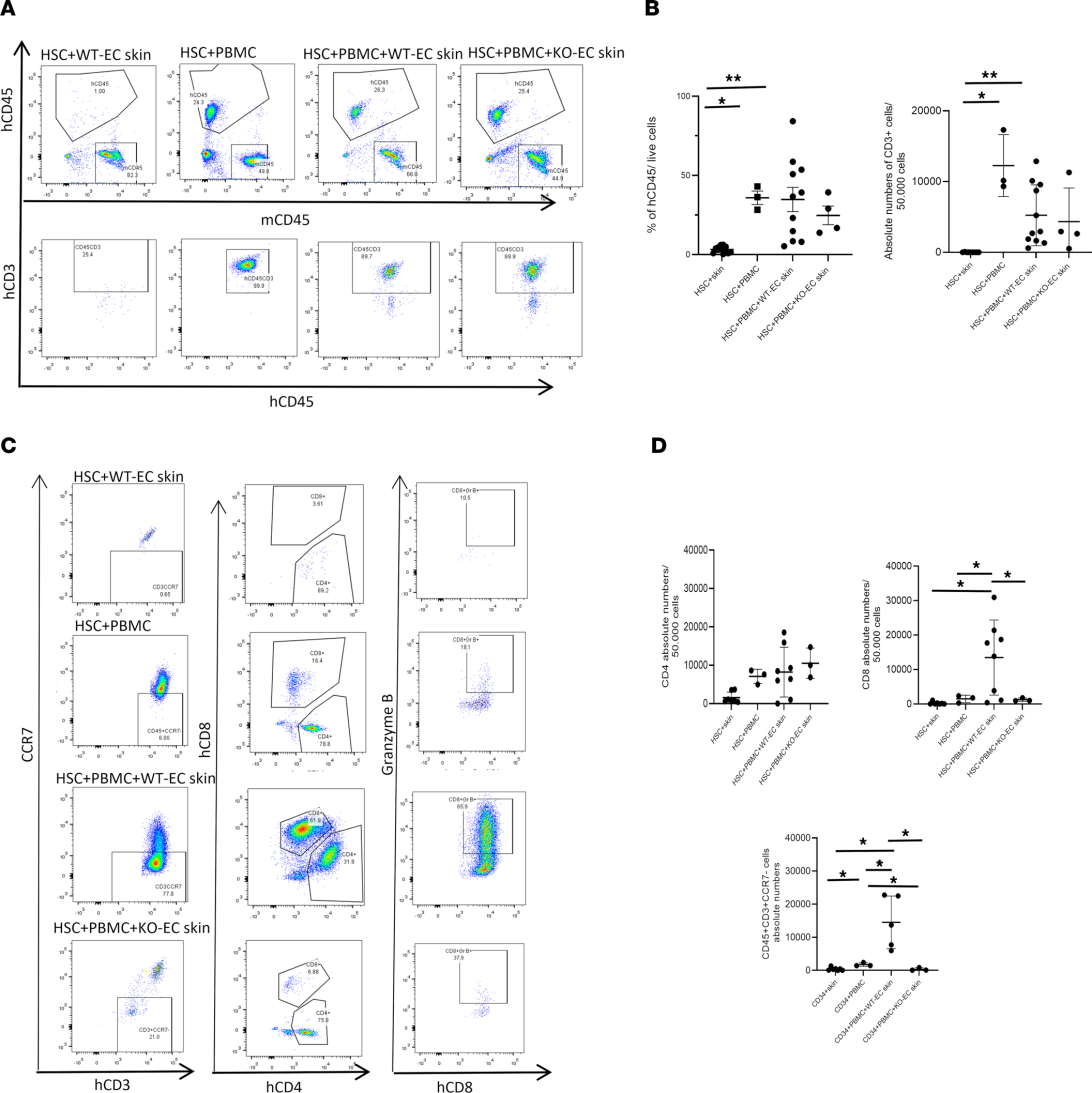
Circulating human cells in skin-grafted MISTRG6 mice. (**A**) Representative analysis of circulating hCD45^+^ and hCD3^+^ cells assessed 3–4 weeks after PBMC injection. (**B**) Statistically significant increase of circulating hCD45^+^ and hCD3^+^ cells shown in HSC+PBMC+WT-EC skin and HSC+PBMC groups (CD45: HSC+skin, *n* = 12, 3.21 ± 2.02 CD45^+^ cells/live cells, vs. HSC+PBMC, *n* = 3, 35.87 ± 7.36 CD45^+^ cells/live cells, *P* = 0.04; HSC+skin vs. HSC+WT-EC skin+PBMC, *n* = 11, 34.79 ± 25.41 CD45^+^ cells/live cells, *P* = 0.01; CD3: HSC+skin, *n* = 9, 13.55 ± 25.07 CD3^+^ cells/50,000 cells, vs. HSC+PBMC, *n* = 3, 12,258.66 ± 4,389.84 CD3^+^ cells/50,000 cells, *P* = 0.04; HSC+skin vs. HSC+PBMC+WT-EC skin, *n* = 11, 5,234.13 ± 4,310.6 CD3^+^ cells/50,000 cells, *P* = 0.01).(**C**) Representative analysis of circulating CD3^+^ cells. Majority of expanded cells in HSC+PBMC+WT-EC skin animals are CCR7^–^, CD8^+^, and granzyme B^+^, indicative of CTL. (**D**) Significant expansion of circulating CD3^+^CCR7^–^ and CD8 cells of HSC+PBMC+WT-EC skin animals shown at week 3–4 after PBMC injection (CD8: HSC+skin vs. HSC+PBMC+WT-EC skin, *n* = 7, 222.1 ± 387.3 vs. *n* = 8, 13,483.87 ± 10,894.9 CD8^+^ cells/50,000 cells, *P* = 0.03, HSC+PBMC vs. HSC+PBMC+WT-EC skin, *n* = 3, 1,468.7 ± 1,068.6 CD8^+^ cells/50,000 cells, *P* = 0.03, and HSC+PBMC+WT-EC skin vs. HSC+PBMC+KO-EC skin, *n* = 3, 1,234 ± 492.1 CD8^+^ cells/50,000 cells, *P* = 0.03; CD3^+^CCR7^–^: HSC+skin, *n* = 7, 387.43 ± 471.84 CD3^+^CR7^–^ cells/50,000 cells vs. HSC+PBMC, *n* = 3, 1747.33 ± 541 CD3^+^CCR7^–^ cells, *P* = 0.03; HSC+skin vs. HSC+PBMC+WT-EC skin, *n* = 5, 14,509.6 ± 7,933.36 CD3^+^ CCR7^–^ cells, *P* = 0.03 or HSC+PBMC vs. HSC+PBMC+WT-EC skin, *P* = 0.03; HSC+PBMC vs. HSC+PBMC+KO-EC skin, *n* = 3, 328.67 ± 445.5 CD3^+^ CCR7^–^ cells, *P* = 0.03, HSC+PBMC+WT-EC skin vs. HSC+PBMC+KO-EC skin, *P* = 0.03). Statistical analysis was performed using Welch’s unpaired *t* test and Benjamini-Hochberg correction for multiple comparisons. *P < 0.05; **P < 0.01. Values are shown as mean ± SD.

We began with analysis of the blood. Mice inoculated as neonates with HSCs and boosted with autologous PBMCs as adults showed a remarkable expansion of circulating CD45^+^ human cells that did not occur in mice treated with either HSC or PBMC inoculation alone. The increase in circulating human cells in animals inoculated with HSCs and boosted with PBMCs was comparable to the increased levels in mice that had been implanted with WT-EC or KO-EC skin before receiving the PBMC boost. Most of these cells were CD4^+^ T cells. In several cases, if reconstitution was delayed and below 10% at week 3 after PBMC injection, week 4 harvest data were added for both HSC+WT-EC skin+PBMC and HSC+KO-EC skin+PBMC groups, and graphic data are presented as pooled data (Figure 2, A and B).

The most striking finding is that the T cell expansion in the skin of mice bearing grafts with WT-ECs but not in mice bearing grafts made with KO-ECs additionally involved a large increase in effector or effector memory (CCR7^–^) CD8^+^ T cells expressing granzyme B, typically characterized as CTL or pre-CTL (Figure 2, C and D).

**Figure 3:**
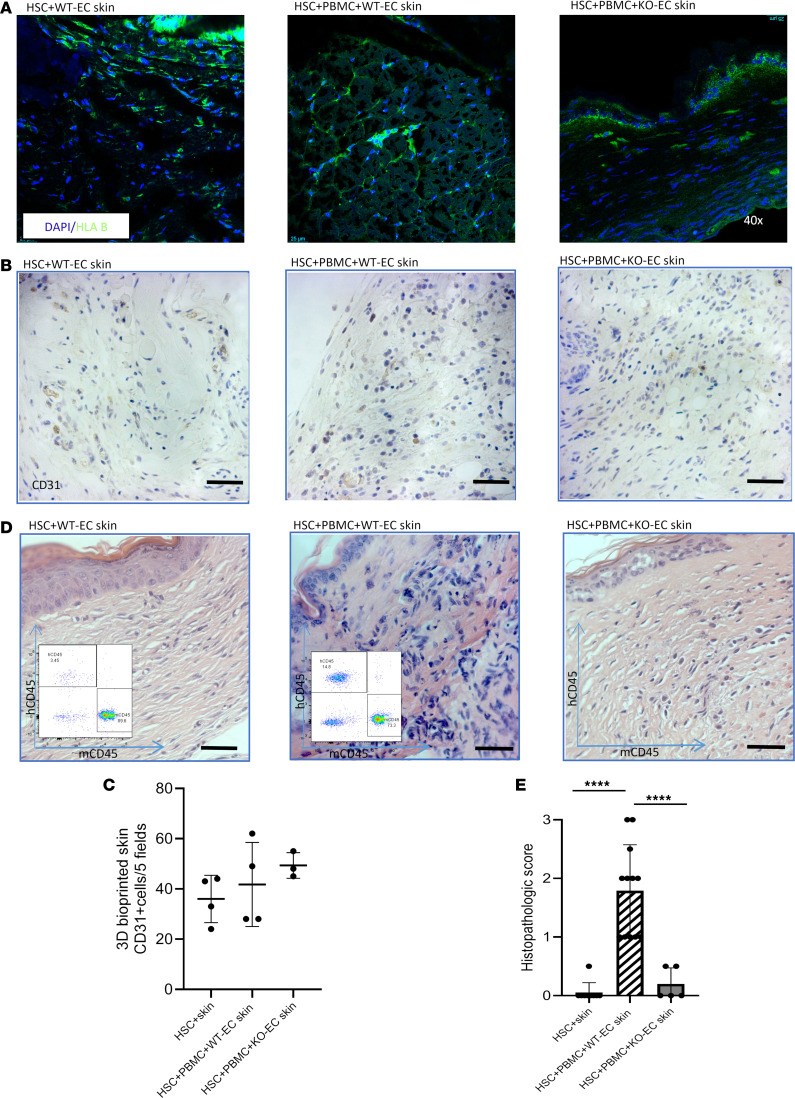
Vitality, vascularization, and rejection assessment of 3D-printed skin grafts. (**A**) Skin graft cells presenting HLA B molecules signalizing vital cells present in both HSC+WT-EC skin and HSC+PBMC+KO-EC skin grafts (although not on KO-ECs, still present on other human cells of skin graft). (**B** and **C**) IHC staining of CD31 shows comparable numbers of human ECs present in HSC+WT-EC skin or WT-EC skin+PBMC– and KO-EC skin+PBMC–implanted skin grafts. (**D**) 3D-printed skin with WT-EC but not KO-EC was rejected (insets show FACS hCD45 dot plots). (**E**) Histopathologic evaluation of tissues showing significant differences between HSC+skin and HSC+PBMC+KO-EC skin mice, in comparison with HSC+PBMC+WT-EC skin (HSC+skin, *n* = 9; histopathologic score, 0.06 ± 0.17 vs. HSC+PBMC+WT-EC skin, *n* = 12; histopathologic score, 1.79 ± 0.7; *P* < 0.0001, and HSC+PBMC+WT-EC skin vs. HSC+PBMC+KO-EC skin, *n* = 5; histopathologic score, 0.2 ± 0.27; *P* < 0.0001). Statistical analysis was performed using Welch’s unpaired t test and Benjamini-Hochberg correction for multiple comparisons. ****P < 0.0001. Means, SDs, and multiple comparison–corrected *P* values are described. Scale bar: 0.5 mm. Values are shown as mean ± SD.

**Figure 4:**
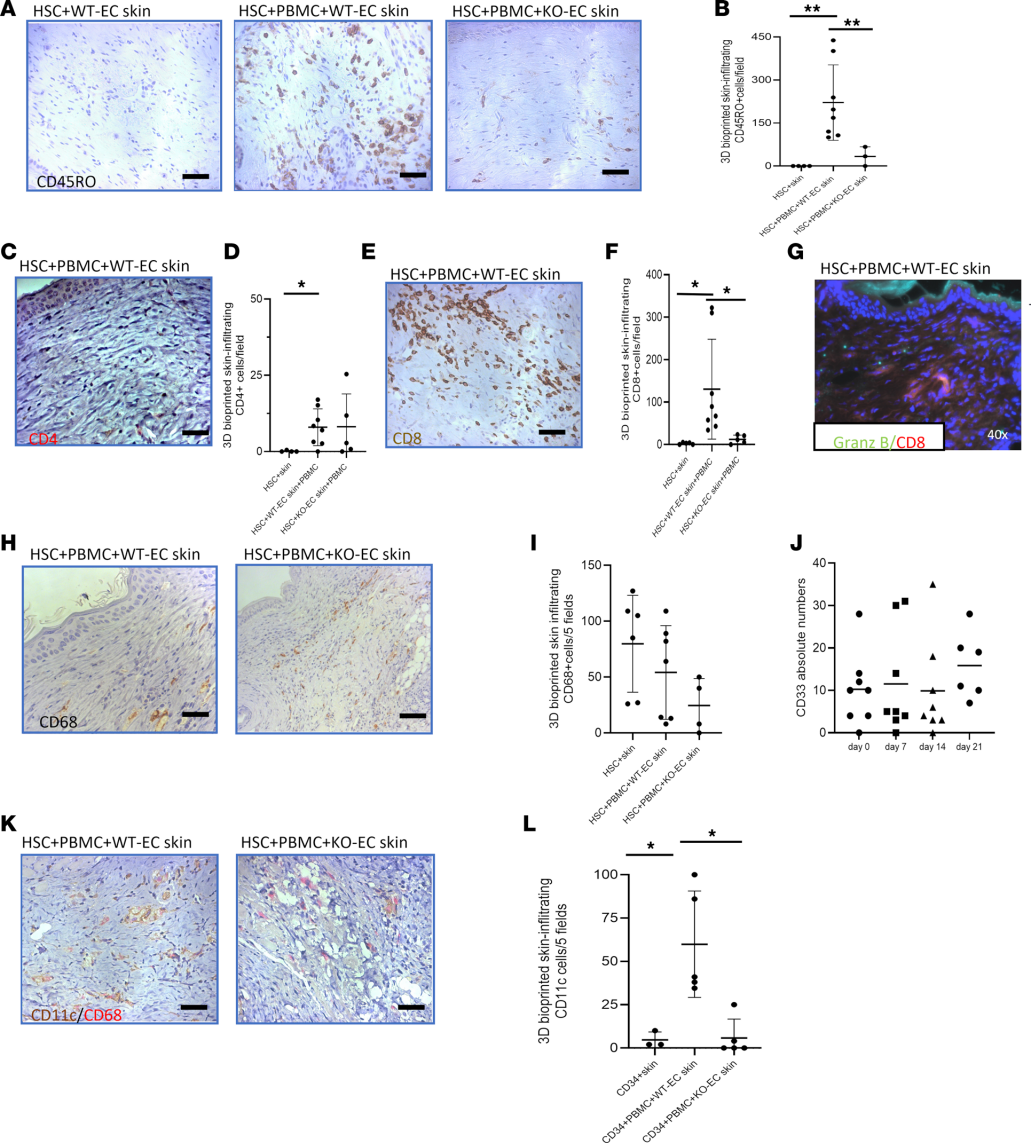
3D-printed skin-infiltrating cells. (**A** and **B**) IHC staining of CD45RO shows massive expansion of memory T cells in WT-EC skin+PBMC– but not in KO-EC skin+PBMC–implanted skin grafts (HSC+skin, *n* = 4, 0 ± 0 cells/field vs. HSC+PBMC+WT-EC skin, *n* = 8, 221.25 ± 131.49 cells/field, *P* = 0.006, HSC+PBMC+WT-EC skin vs. HSC+PBMC+KO-EC skin, *n* = 3, 33.67 ± 33.5 cells/field, *P* = 0.007). (**C** and **D**) Similar presence of CD4 lymphocytes found in WT+EC skin+PBMC or HSC+PBMC+KO-EC skin grafts (HSC+skin, *n* = 4, 0.12 ± 0.25 cells/field vs. HSC+PBMC+WT-EC skin *n* = 8, 7.98 ± 6.04, *P* = 0.02). (**E** and **F**) Massive expansion of CD8 lymphocytes in the HSC+PBMC+WT-EC skin group as compared with the HSC+skin or HSC+PBMC+KO-EC skin groups (HSC+skin, *n* = 5, 2.4 ± 2.3 cells/field vs. HSC+PBMC+WT-EC skin, *n* = 8, 130.46 ± 117.73 cells/field, *P* = 0.04; HSC+PBMC+WT-EC skin vs. HSC+PBMC+KO-EC skin, *n* = 5, 12.18 ± 10.06 cells/field, *P* = 0.04). (**G**) 3D-printed skin-infiltrating CD8 lymphocytes show Granzyme B positivity. (**H** and **I**) IHC staining of CD68 shows infiltration of skin grafts by macrophages present in all 3 PBMC+skin, WT-EC skin+PBMC and KO-EC skin+PBMC implanted skin grafts. (**J)** Stable circulating human myeloid cells (CD33^+^ cells) at weekly assessment for 4 weeks. (**K** and **L**) CD11c/CD68 double staining revealed infiltrating dendritic cell populations in WT-EC but not KO-EC skin mice (CD68: PBMC+skin, *n* = 6, 79.83 ± 43.41 cells/field; WT-EC skin+PBMC, *n* = 7, 54.14 ± 41.69 cells/field and KO-EC skin+PBMC, *n* = 4, 24.5 ± 24.24 cells/field; CD11c: HSC+skin, *n* = 3, 4.66 ± 4.62 cells/field vs. HSC+PBMC+WT-EC skin, *n* = 5, 59.9 ± 30.7 cells/field, *P* = 0.02, HSC+PBMC+WT-EC skin vs. HSC+PBMC+KO-EC skin, *n* = 5, 5.8 ± 10.87 cells/field, *P* = 0.02). Statistical analysis was performed using Welch’s unpaired *t* test and Benjamini-Hochberg correction for multiple comparisons. *P < 0.05; **P < 0.01. Means, SDs, and multiple comparison–corrected *P* values are described. Original magnification, ×40 (**G**). Scale bar: 0.5 mm. Values are shown as mean ± SD.

We next examined the bioprinted skin grafts. All of the skin grafts showed HLA-B^+^ cells, both indicating their human origin and confirming that grafts constructed with KO-ECs still expressed HLA antigens on other cell populations (Figure 3A). We also found a comparable level of human CD31-lined vessels in both types of grafts (Figure 3, B and C; HSC+WT-EC skin (*n* = 4, 36 ± 9.41 cells/5 fields, mean ± SD), WT-EC skin+PBMC (*n* = 4, 41.75 ± 16.74 cells/5 fields, mean ± SD) or KO-EC skin+PBMC (*n* = 3, 49.33 ± 5.13 cells/5 fields, mean ± SD), confirming that KO-ECs retained their capacity to vascularize the constructs. Both WT-EC and KO-EC skin grafts were also infiltrated by mouse EC-lined vessels (Supplemental Figure 1A; supplemental material available online with this article; https://doi.org/10.1172/jci.insight.201946DS1). In addition, skin grafts made with WT-ECs were infiltrated by human leukocytes within 3–4 weeks after the boost, largely in a perivascular distribution, and showed histologic evidence of injury. In contrast, skin grafts made with KO-ECs showed minimal evidence of injury (Figure 3, D and E) and developed only minimal levels of infiltration by human CD45RO lymphocytes (Figure 4, A and B). Human ECs in the WT-EC–rejecting grafts showed upregulation of E selectin, consistent with a human cytokine response, but KO-EC skins lacked expression of E selectin (Supplemental Figure 1B).

We then focused on the nature of the human leukocytic infiltrates in the grafts containing WT-ECs. Consistent with our analysis of circulating cells, the majority of the infiltrate consisted of CD8^+^ T cells that expressed granzyme B (Figure 4, C–G). Such cells were absent in the grafts containing KO-ECs (Figure 4, F and G). Infiltrates in the rejecting grafts also included CD68^+^ macrophages and CD68^–^CD11c^+^ cells, which we tentatively identified as dendritic cells. Grafts with KO-EC cells showed infiltrating CD68^+^ macrophages but lacked CD11c^+^ cells (Figure 4, H, I, K, and L), whereas mice receiving human HSC, WT-EC, or KO-EC skin grafts and human PBMCs showed stable numbers of circulating human myeloid cells weekly for 3 weeks after PBMC injection (Figure 4J).

**Figure 5:**
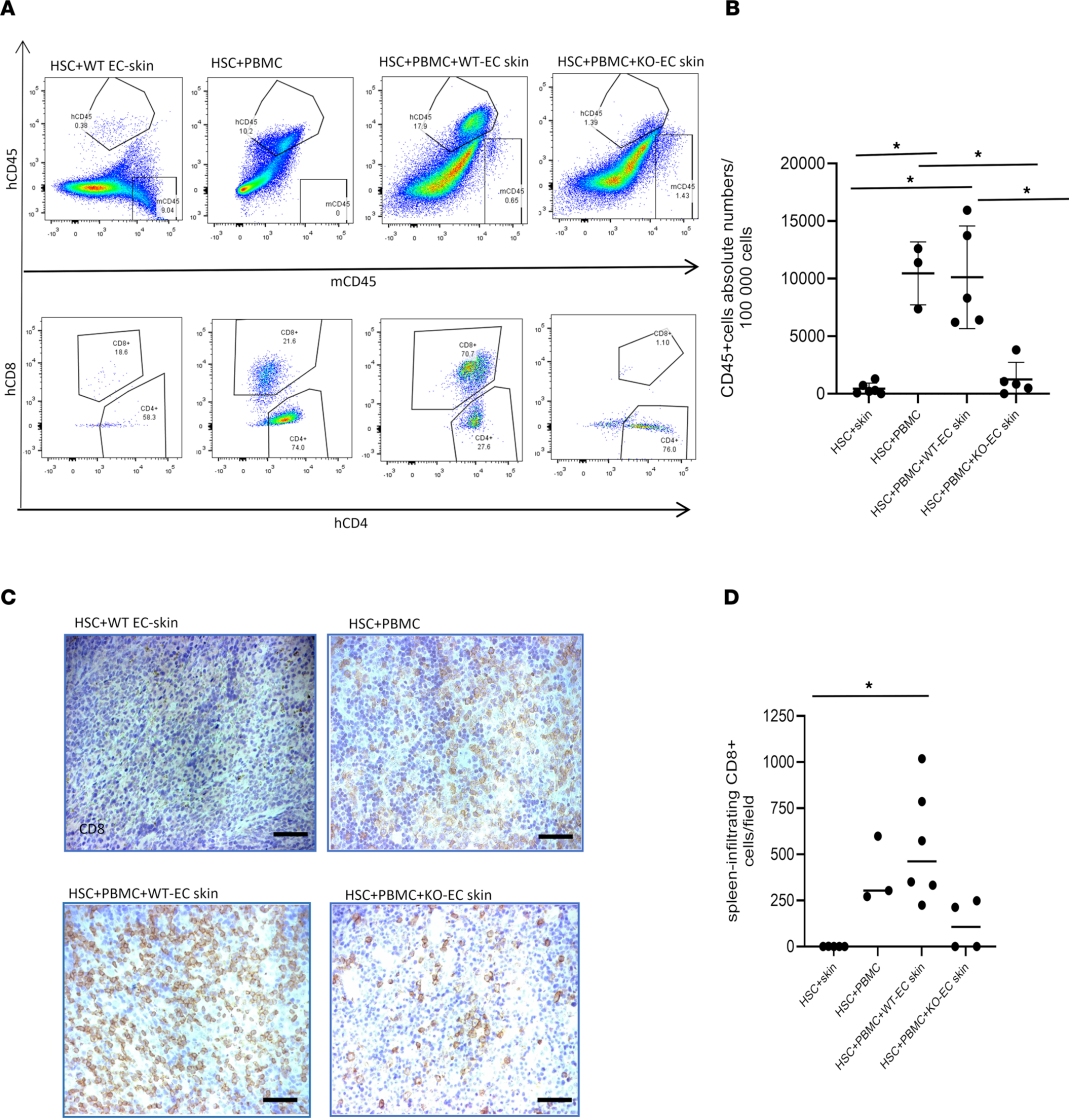
Characterization of spleen-infiltrating human cells. (**A**) Comparison of infiltrating human cells by hCD45 and hCD4/CD8 staining. hCD45 staining shows minimal hCD45 cell infiltration of spleens of HSC+PBMC+KO-EC skin animals. Consistent with circulation and skin grafts, robust expansion of CD8 lymphocytes was seen in HSC+PBMC+WT-EC skin animals, whereas low infiltration was seen in HSC+PBMC+KO-EC skin mice. (**B**) Comparison of circulating human leukocytes shows statistical significant hCD45 leukocytes in the HSC+WT-EC skin+PBMC group as compared with the HSC+skin or HSC+KO-EC skin+ PBMC groups. (**C** and **D**) IHC staining reveals expansion of CD8 lymphocytes in HSC+PBMC+WT-EC skin mice (hCD45: HSC+skin, *n* = 6, 434 ± 489.2 cells/field vs. HSC+PBMC, *n* = 3, 10,456.6 ± 2,738.98 cells/field, *P* = 0.03; HSC+skin vs. HSC+PBMC+WT-EC skin, *n* = 5, 10,120.46 ± 4,456.34 cells/field, *P* = 0.02; HSC+PBMC+WT-EC skin vs. HSC+PBMC+KO-EC skin, *n* = 5, 1,242.4 ± 1,485.17 cells/field, *P* = 0.03; HSC+PBMC vs. HSC+PBMC+WT-EC skin, *P* = 0.03; CD8: HSC+skin, *n* = 5, 1 ± 0.71 cells/field vs. HSC+PBMC+WT-EC skin, *n* = 6, 547.67 ± 305.78 cells/field, *P* = 0.04; mean ± SD). Statistical analysis was performed using Welch’s unpaired *t* test and Benjamini-Hochberg correction for multiple comparisons. *P < 0.05. Means, SDs, and multiple comparison–corrected *P* values are described. Scale bar: 0.5 mm.

Finally, to assess the systemic nature of the immune response against the skin, we examined the spleens. The spleens of the animals with rejecting skin grafts were noticeably enlarged (0.8–1.1 cm vs. 2.2–2.4 cm). Analysis of the spleens by both histology and flow cytometry confirmed the presence of hCD45^+^ infiltration and large numbers of human CD8^+^ T cells in WT-EC skin grafts (Figure 5).

**Figure 6:**
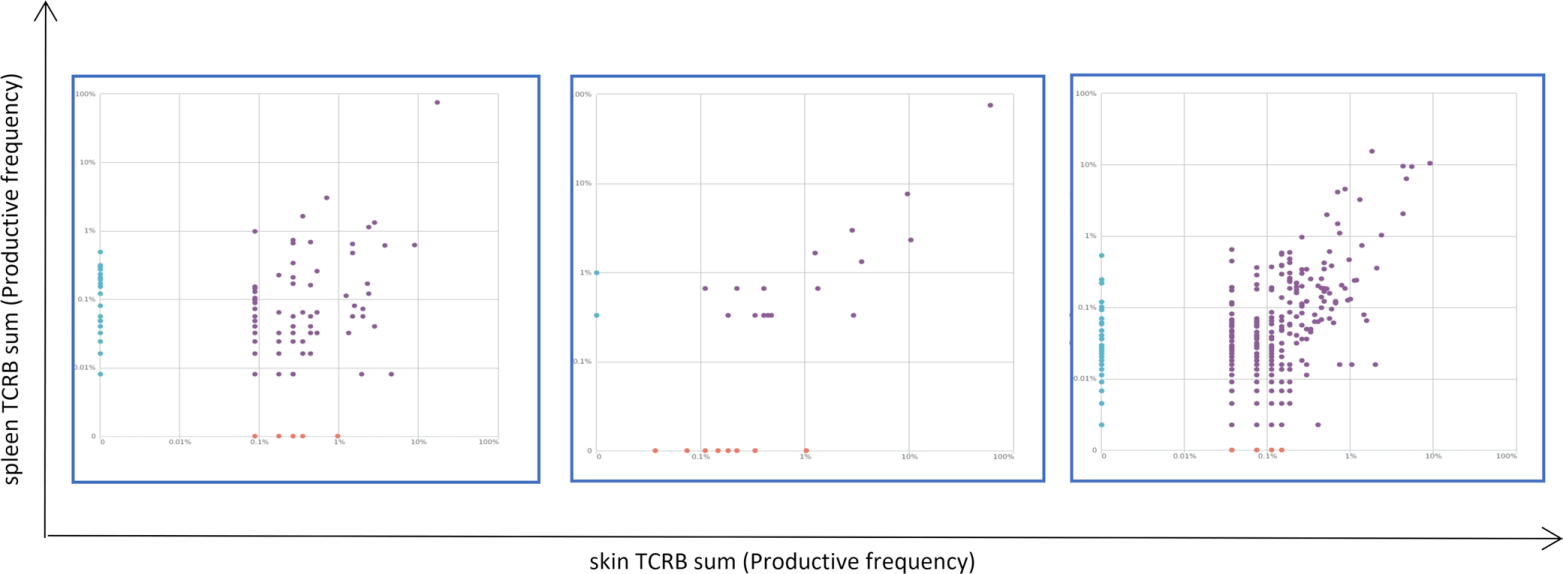
Immunosequencing of the T cell receptor repertoire of skin and matching spleen samples. High-throughput sequencing of the β chain of T cell receptors. *x* and *y* axes show total amount of productive TCR β sequences in the skin or spleen samples with 1–2 immunodominant clones in 2 of 3 pairs of investigated samples.

Some animals receiving grafts with KO-ECs also showed splenic enlargement but did not show expanded human CD8^+^ T cells. Involvement of the spleen raised the issue of whether the CD8^+^ infiltrates were evidence of a systemic alloresponse against the graft or xenogeneic graft-versus-host disease possibly triggered by the alloresponse. To address this question, we analyzed TCRβ sequences of the human T cells in the skin and spleens of 3 pairs of the mice showing evidence of graft rejection. Strikingly, there was a large number of shared clones, including a few highly immunodominant clones in 2 of the 3 pairs, but the sequence of the immunodominant clones were not the same among the 3 animals (Figure 6). We interpret this as strong evidence for an alloresponse being systemic; the involvement of mouse tissues in addition to the skin graft is suggestive of semidirect recognition triggered by shedding of EC microparticles bearing intact MHC molecules into the circulation.

## Discussion

Rodent models of transplantation are widely used for studying allograft rejection mechanisms, but many features of rodent immunology differ remarkably from clinical experience. Human immune system mice appear to offer a possible bridge in which human cell-mediated rejection can be studied in a small animal model (22). Natural or synthetic human skin grafts in human immune system mice offer a way to analyze the role of blood vessels in the human allogeneic response. We and others have extensively exploited this approach, but an often overlooked limitation is the incompleteness of the engrafted human immune system. Specifically, transplant rejection in the clinic not only involves direct T cell recognition of graft endothelium, but also involves innate immune cells, either as effector cells or as mediators of indirect or semidirect recognition; these cells are missing in the most commonly used mouse models. Here, we have introduced a model that addresses these prior limitations and reveals the importance of ECs in initiating rejection.

The MISTRG6 mouse has been engineered to allow development of innate myeloid and lymphocyte populations. As such, these mice are invaluable in studying responses to infection. Transplantation raises another issue, namely that a large element of the immune response to allografts is based on cross-reactive memory acquired from infections, and this cannot be easily generated in animals without a high incidence of fatality. We have addressed this problem by inoculated neonatal MISTRG6 mice with adult HSCs and then reinoculating the same animals upon maturity with PBMCs, containing alloreactive effector memory T cells, from the same blood donor. Our exact protocol, developed from experience, is to place a skin graft 3 weeks before the administration of the PBMCs. This timing allows blood vessels in the graft to connect with host vessels, mimicking the establishment of surgical anastomoses in solid organ transplantation.

A second feature of our advanced model is that it uses synthetic rather than natural skin. This has two advantages. First, the knockin of IL-6 in the MISTRG6 model induces a nonphysiological expansion of resident leukocytes in normal human skin that obscures transplant responses (26). Second, and more importantly, it allows us to control the composition of the skin substitute. An important feature of our model is that the ECs, pericytes, fibroblasts, and keratinocytes are all acquired from a single donor. To assemble these cell populations in a manner recapitulating key features of normal skin, we used 3D printing to separate the dermal from epidermal layer. This approach can be generalized for future work to incorporate other cell types, such as leukocytes, melanocytes, or cells of skin adnexa.

There are two key findings of our study. First, we can generate a rejection response that involves human myeloid cells as well as lymphocytes. Indeed, the myeloid compartment appears critical in triggering a T cell response, as judged by T cell expansion in the blood and spleen as well as the graft in animals receiving HSCs as well as PBMCs. The absence of myeloid cells at first seems to contradict our prior work in which adoptively transferred PBMCs alone could cause rejection in C.B-17 SCID/bg mice (18). The simple explanation is that we had to introduce at least a hundred-fold more PBMCs or purified T cells to produce that result than we used in the current experiment. In the absence of myeloid cells, the T cells in the MISTRG6 animals are too few and simply fail to expand. Second, we show that it is EC presentation of antigen rather than increased vascular perfusion that leads to rejection. This conclusion has major implications for tissue engineering using allogeneic cells, namely that (a) ECs are a critical cell type for initiation of rejection and (b) MHC molecule expression on other cell types may be less critical. We note that this finding is consistent with the clinical experience using tissue engineered cellularized skin, in which allogeneic fibroblasts and keratinocytes do not activate a recipient allogeneic rejection response.

While this study offers a model with advances compared with that used in prior human immune system mouse studies, there remain limitations. First, we do not know if ECs need to lack class I or class II or both classes of MHC molecules to avoid rejection. Our prior work suggested class I molecules were more important, but both played a role (20).

Second, the systemic nature of the alloresponse in this study suggests a role for shedding of intact MHC molecules and semidirect presentation. This issue warrants further investigation. Third, while the protection from rejection when ECs lack both class I and class II MHC molecules appears fairly complete, we stopped the experiment at 3–4 weeks after PBMC inoculation because the mice that received grafts with WT-ECs became fragile as circulating T cell numbers grow very large. In future work, we can assess if the animals receiving grafts containing ECs lacking MHC molecules — but where MHC expression is retained by other cell types — will reject their grafts eventually (by indirect presentation) or if modulation of EC immunogenicity is sufficient to eliminate rejection by tolerizing the immune response. Finally, we have not explored the role of alloantibody in graft rejection, an issue that also warrants further study. Despite these limitations, the present study significantly closes the gap between preclinical models and clinical experience. It also suggests that the recipient immune responses to tissue engineered grafts, like that to allogeneic organs, is more dependent upon some cell types, such as ECs, than on others.

## Methods

### Sex as a biological variable.

Cells were isolated from placenta, cord blood, and foreskin. Use of foreskin, as a source of fibroblasts and keratinocytes, limited cell sampling to male babies.

Both female and male mice were utilized in the 3D-printed skin implantation experiments, with no differences observed.

### Antibodies and other reagents.

Antibodies used in these studies are described in Supplemental Table 1.

### 3D-printed skin graft preparation.

Anonymized tissue donors for 3D-bioprinted skin production were acquired through the Yale University Reproductive Sciences Biobank from discarded tissues with informed consent. Cells involved in the skin graft printing were isolated, cultured and characterized as described, as were the composition of the bioinks the 3D printing process and surgical engraftment (14). In brief, human pericytes were isolated from discarded placenta, endothelial colony-forming cells (ECFCs) were isolated from cord blood, fibroblasts and keratinocytes were isolated from discarded male foreskins, and each cell type was expanded in vitro. All 4 cell types used in each graft were obtained from a single male infant donor, and a total of 3 different donors were used over the course of these studies and gave similar results. Where indicated, unmodified ECFC-derived ECs (WT-ECs) were replaced with ECFC-derived ECs from the same donor that had been modified by CRISPR/Cas9, as described to ablate expression of class I and II MHC molecules (20). 3D bioprinting was performed with a BioX bioprinter (CELLINK) as described. The only change in the process from our description was that the dermal bioink was introduced into a sterile nonwoven polyglycolic acid mesh (Confluent Medical Technologies Inc.) to delay contraction after implantation, allowing the dermal cells more time to synthesize extracellular matrix. As we described previously, the mesh is absorbed by 3 weeks in the absence of human leukocytes, with minimal inflammation and no effect on graft cell viability (27).

### Animals.

MISTRG6 mice were engineered by a human/mouse homolog gene-replacement strategy to provide physiological expression of human M-CSF (monocytes and tissue macrophage development), GM-CSF/IL-3 (lung alveolar macrophages), SIRPα (tolerance of macrophages to human cells), ThPO (hematopoiesis and platelets), and IL-6 (improved engraftment and antibody responses) on a Rag2/**γ** common chain–deleted background (22, 23). Availability of mice was limiting, and both males and females were used interchangeably with no obvious differences in outcomes.

### Experimental outline.

The general experimental protocol used is shown in Figure 1. MISTRG6 neonates were irradiated, anti-CD3 antibody–treated, and injected intrahepatically within 24–48 hours after birth with human adult CD34^+^ HSCs obtained from a cryopreserved, discarded, and anonymized GM-CSF–mobilized donor leukapheresis collection. HSCs were isolated by density gradient centrifugation (Lymphoprep, StemCell Technologies) followed by immunomagnetic selection (EasySep Human CD34 Positive Selection Kit, StemCell Technologies) and assessed by flow cytometry for purity. Isolations contained over 93% CD34^+^ cells. Once the animals showed stable levels of circulating human cells, confirmed by flow cytometry (typically at 10 weeks), 3D-bioprinted skin grafts, containing cells allogeneic to injected CD34^+^ cells, were implanted on dorsal part of MISTRG6 mice. Healing of the skin grafts after surgery was allowed to occur for 2 weeks. At that point, mice were injected with autologous PBMCs to the HSCs. Mice were monitored for signs of rejection and harvested at various time points (day 17, day 23, day 28–34). Control groups in some experiments omitted HSC injection, omitted PBMC injection, omitted skin grafting, or omitted injection, as follows: (a) mice receiving human adult HSCs as neonates and human PBMCs from the same individual as adults without skin grafting; (b) mice receiving a 3D-printed human skin grafts made with WT-ECs as adults in the absence of human hematopoietic cells; (c) mice receiving human HSCs as neonates and a 3D-printed human skin with WT-ECs from a source allogeneic to the HSCs as adults without a PBMC boost; (d) mice receiving human HSCs as neonates and a 3D-printed human skin with KO-ECs from a source allogeneic to the HSCs as adults; and (e) mice receiving 3D-printed human skin with WT-ECs as adults and PBMCs from a donor allogeneic to the skin cell source 3 weeks later but without neonatal inoculation with human HSCs. These groups constitute our controls, and key findings are summarized in Supplemental Table 2 and Supplemental Figure 2. Overall, we analyzed 47 MISTRG6 mice using 1 leukocyte donor and 3 skin donor collections. Not every experiment contained all of the controls, as we were limited by litter size and availability of MISTRG6 animals.

### Flow cytometry.

Flow cytometry was performed on LSR2 or Fortessa instruments (both Becton Dickinson) to characterize cell populations used for 3D bioprinting (as described in ref. 14) and for characterization of circulating cells and cells extracted from tissue samples at the time of sacrifice. Circulating cells were assessed for species origin (using mouse and human CD45) as well as for human cell types (CD3 T cells, CD19 B cells, NKp46 NK cells, CD33 myeloid cells) throughout the experiment, as indicated in Figure 1. Cells extracted from skin grafts and from spleens were analyzed at the time of mice harvest for CD3 T cells, subpopulations (CD4 and CD8) and effector memory cells (CCR7 negative) CD8^+^ T cells were additionally assessed for granzyme B indicative of cytolytic potential. Flow cytometric data were analyzed by Flow Jo software (BD Biosciences).

### Morphological analysis of tissue samples.

H&E staining and immunohistochemical and immunofluorescent staining were performed on flash-frozen cryosections and FFPE sections as indicated in each panel (14). The H&E-stained sections were used to determine the extent of rejection in skin samples (day 17, day 23, days 28–34) and were assessed. The histopathological score of skin transplantation rejection was determined based on the following scoring system: 0, no graft inflammation, normal vessels and ECs lining; 1, infiltrating cells (inflammation), no vascular damage; 2, infiltrating cells (inflammation) and signs of vascular damage; and 3, infiltrating cells (inflammation) and profound vascular damage, assessed by a dermatopathologist blinded to the treatment groups.

The presence and viability of bioprinted and implanted dermal graft cells were assessed by HLA-B staining. Skin grafts were evaluated for the following markers: CD56, CD68, CD11c, CD45RO, human and mouse CD31, CD3, CD4, CD8, granzyme B, or E selectin. All immunofluorescent samples were analyzed by EVOS (Thermo) or confocal microscopy (Stellaris 5, Leica) and IHC samples by Zeiss Axiovert (Supplemental Table 1, list of antibodies).

### TCR sequencing.

Comprehensive analysis of the TCR receptor β chain was performed by Adaptive Biotechnologies to assess the clonality of infiltrating lymphocytes in skin grafts and spleens of 3 different skin donors, all with the same HSC and PBMC donor (FFPE curls, 50 �m). The sequencing repertoire data were analyzed by immunoSEQ 3.0 Analyzer (Adaptive Biotechnologies) and a freely available VDJTools software package for programming language R (28).

### Statistics.

Statistical analysis was performed by using 2-tailed Welch’s unpaired *t* test by GraphPad Prism 10.3.0 software. The data are presented as mean ± SD, and Benjamini-Hochberg correction was applied for multiple comparisons. Means, SDs, and multiple comparison–corrected *P* values are described in the figure legends, and *P* values of less than 0.05 were considered significant.

### Study approval.

Deidentified human tissues for 3D-bioprinted skin production were acquired through the Yale University Reproductive Sciences Biobank from discarded tissues with informed consent. Mouse experiments were performed in compliance with protocols approved by the Yale Institutional Animal Care and Use Committee.

### Data availability statement.

All data supporting the findings of this study, including the Supporting data values file, are available online at Yale Dataverse (https://dataverse.yale.edu/previewurl.xhtml?token=8d3436db-bfab-4ac1-bcaa-fc8986aab1eb).

## Authors contributions

Concept and design: ZT, RAF, WMS, and JSP. Acquisition of materials, annotation, and interpretation of data: all authors. Experiments performed: ZT, ES, LQ, and GD. Acquisition of mice: ES and RAF. Data analysis: ZT, JMM, WMS, and JSP. Drafting of the manuscript: ZT, WMS, and JSP. Critical editing of the manuscript: all authors.

## Conflict of interest

WMS is a cofounder, a member of the board of directors, and a consultant to Xanadu Bio. He is also a consultant to Stradefy Biosciences, Cranius, and CMC Pharma.

## Funding support

This work is subject to the NIH Public Access Policy. Through acceptance of this federal funding, the NIH has been given a right to make the work publicly available in PubMed Central.

- National Cancer Institute Cancer Center Support Grant P30 CA016359 supported the Yale Flow Cytometry Core.
- National Institute of Allergy and Infectious Diseases grant 1R01HL169238-01A1.

## Supplementary Material

Supplemental data

Supporting data values

## Version 1 04/22/2026

Electronic publication

## References

[1] Janson KD (2025). Strategies for the vascular patterning of engineered tissues for organ repair. Nat Biomed Eng.

[2] Eming SA (2014). Wound repair and regeneration: mechanisms, signaling, and translation. Sci Transl Med.

[3] Dixit S et al (2017). Immunological challenges associated with artificial skin grafts: available solutions and stem cells in future design of synthetic skin. J Biol Eng.

[4] Burke JF (1981). Successful use of a physiologically acceptable artificial skin in the treatment of extensive burn injury. Ann Surg.

[5] Shevchenko RV (2010). A review of tissue-engineered skin bioconstructs available for skin reconstruction. J R Soc Interface.

[6] Sierra-Sanchez A (2021). Cellular human tissue-engineered skin substitutes investigated for deep and difficult to heal injuries. NPJ Regen Med.

[7] Serra RA (2017). Skin grafting for the treatment of chronic leg ulcers - a systematic review in evidence-based medicine. Int Wound J.

[8] Griffiths M (2004). Survival of Apligraf in acute human wounds. Tissue Eng.

[9] Mastrogiacomo M (2022). Innovative cell and platelet rich plasma therapies for diabetic foot ulcer treatment: the allogeneic approach. Front Bioeng Biotechnol.

[10] Allie DE (2004). Novel treatment strategy for leg and sternal wound complications after coronary artery bypass graft surgery: bioengineered Apligraf. Ann Thorac Surg.

[11] Pierce RW (2017). Endothelial cell function and dysfunction in critically ill children. Pediatrics.

[12] Pober JS (2003). Immunopathology of human T cell responses to skin, artery and endothelial cell grafts in the human peripheral blood lymphocyte/severe combined immunodeficient mouse. Springer Semin Immunopathol.

[13] Schechner JS (2003). Engraftment of a vascularized human skin equivalent. FASEB J.

[14] Baltazar T (2020). Three dimensional bioprinting of a vascularized and perfusable skin graft using human keratinocytes, fibroblasts, pericytes, and endothelial cells. Tissue Eng Part A.

[15] Abrahimi P et al (2015). Blood vessels in allotransplantation. Am J Transplant.

[16] Lakkis FG, Lechler RI (2013). Origin and biology of the allogeneic response. Cold Spring Harb Perspect Med.

[17] Espinosa JR (2016). Memory T cells in organ transplantation: progress and challenges. Nat Rev Nephrol.

[18] Shiao SL (2007). Human effector memory CD4+ T cells directly recognize allogeneic endothelial cells in vitro and in vivo. J Immunol.

[19] Carnel N (2023). Pathways of antigen recognition by T cells in allograft rejection. Transplantation.

[20] Merola J (2019). Progenitor-derived human endothelial cells evade alloimmunity by CRISPR/Cas9-mediated complete ablation of MHC expression. JCI Insight.

[21] Kenney LL (2016). Humanized mouse models for transplant immunology. Am J Transplant.

[22] Sefik E (2022). A humanized mouse model of chronic COVID-19. Nat Biotechnol.

[23] Rongvaux A (2014). Development and function of human innate immune cells in a humanized mouse model. Nat Biotechnol.

[24] Ford ML, Kirk AD, Larsen CP (2009). Donor-reactive T-cell stimulation history and precursor frequency: barriers to tolerance induction. Transplantation.

[25] Sefik E (2025). Engineering mice to study human immunity. Annu Rev Immunol.

[26] Odell ID (2023). IL-6 trans-signaling in a humanized mouse model of scleroderma. Proc Natl Acad Sci U S A.

[27] Baltazar T (2023). 3D bioprinting of an implantable xeno-free vascularized human skin graft. Bioeng Transl Med.

[28] Shugay M (2015). VDJtools: unifying post-analysis of T cell receptor repertoires. PLoS Comput Biol.

