# Supplementary material for: Endothelial MHC expression is required to initiate T cell–mediated rejection of 3D-printed skin grafts
Source: JCI Insight. 2026 Apr 22;11(8):e201946. doi: 10.1172/jci.insight.201946 (PMC13135413; doi:10.1172/jci.insight.201946)
Supplement: Supplemental data [file jciinsight-11-201946-s172.pdf]

Supplementary Figure 1.

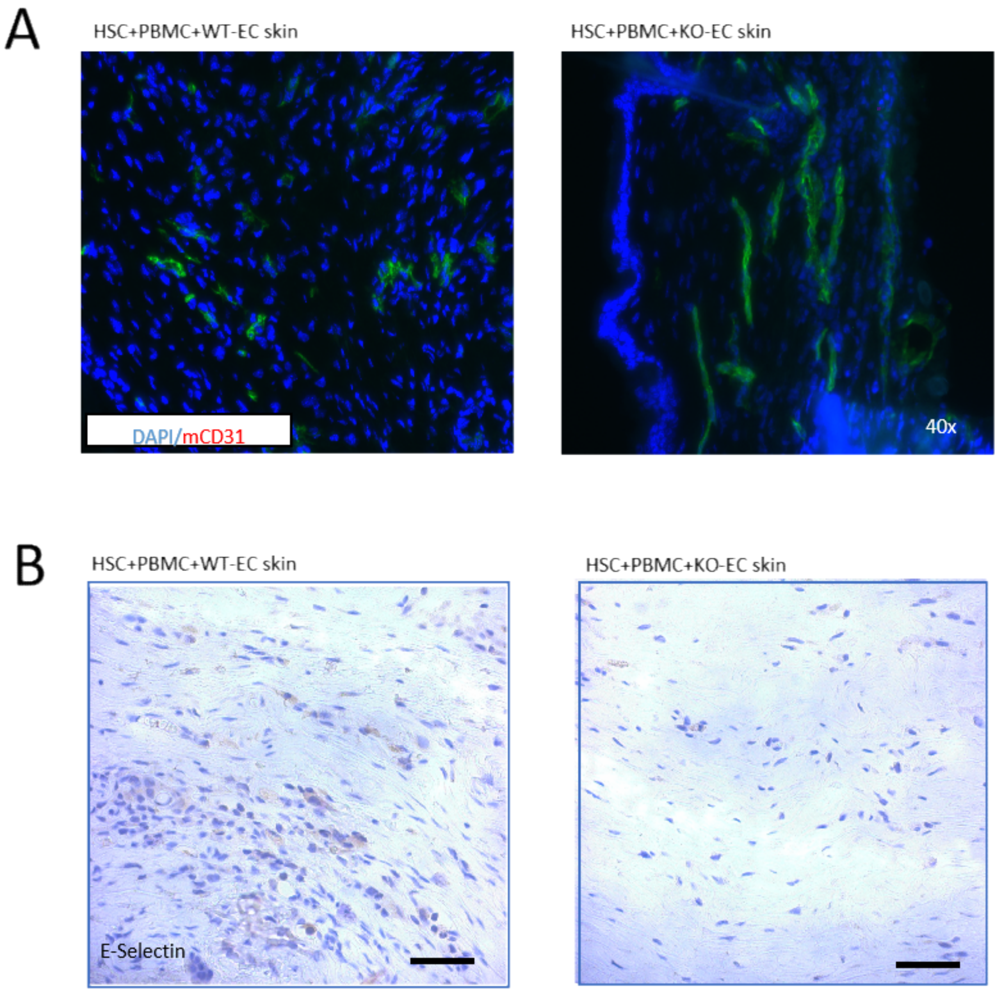

Supplementary Figure 1. Further characterization of implanted 3D-printed skin grafts

A) Staining of mCD31 infiltrating human part of skin shows similar presence of mice endothelial cells in the HSC+PBMC+WT-EC skin or HSC+PBMC+KO-EC skin grafts indicating undamaged mouse endothelial cells.

B) Presence of E-Selectin staining in HSC+PBMC+WT-EC tissues whereas no activation marker positivity in HSC+PBMC+KO-EC skin grafts.

Supplementary Figure 2.

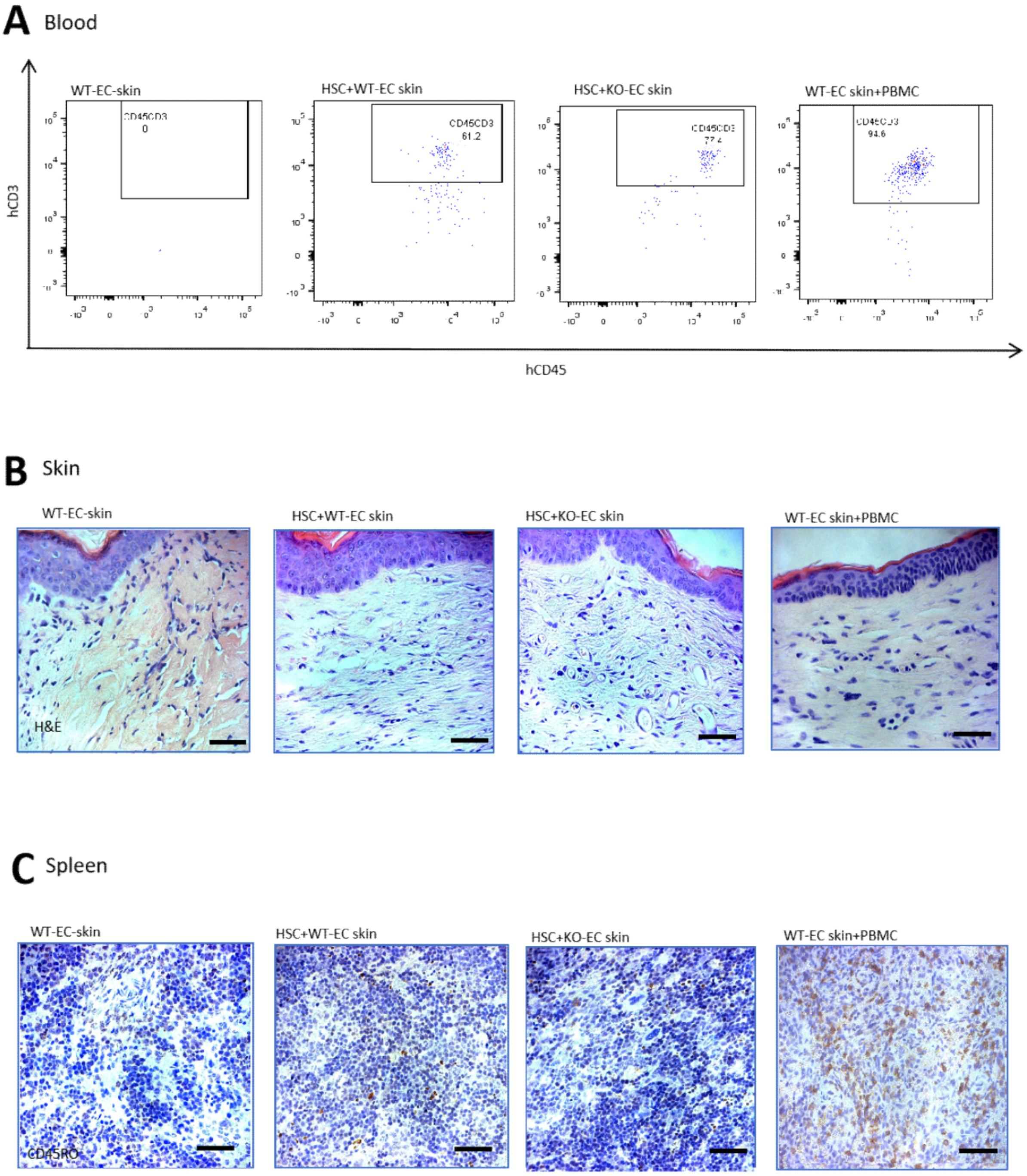

Supplementary Figure 2. Blood, skin and spleen samples of control groups (2)-(5) (described in details in the Methods section).

A) Dot plots of circulating human CD45/CD3 cells in (2) WT-EC skin mice, (3) HSC+WT-EC skin mice, (4) HSC+KO-EC skin mice and (5) WT-EC-skin+PBMC mice.

A) H&E staining of 3D-printed skins grafts of (2) WT-EC skin mice, (3) HSC+WT-EC skin mice, (4) HSC+KO-EC skin mice and (5) WT-EC-skin+PBMC mice.

B) CD45RO staining of spleens of (2) WT-EC skin mice, (3) HSC+WT-EC skin mice, (4) HSC+KO-EC skin mice, (5) WT-EC-skin+PBMC mice indicating infiltration of spleens by human memory T cells in WT-EC-skin+PBMC mice only.

Supplementary Table 1. List of used antibodies/dyes

Antigen	Fluorophore	Clone	Cat #	Source
mCD45	FITC	I3/2.3	147710	Biolegend
CD45	PB	2D1	368540	Biolegend
CD4	FITC	A161A1	357406	Biolegend
CD33	APC	P67.6	366606	Biolegend
CD19	APC Cy7	HIB19	302218	Biolegend
CD335 (NKp46)	PE Cy7	9E2	331916	Biolegend
CD66b	PE	6/40c	392904	Biolegend
CD8	APC	SK1	344722	Biolegend
CD8	Spark Plus UV 395	RPA--T8	301080	Biolegend
CCR7	PE	G043H7	353204	Biolegend
CCR7	APC	G043H7	353213	Biolegend

Granzyme B	PE/Dazzle 594	QA16A02	372216	Biolegend
CD4	APC Cy7	OKT4	317418	Biolegend
CD25	PE	M-A251	557138	Becton Dickinson
CD11c	APC	L161	331523	Biolegend
HLA ABC	FITC	W6/32	311404	Biolegend
HLA DR	AF647	L243	307622	Biolegend
HLA DR	AF647	LN3	327012	Biolegend
CD8	Dylight 550	C8/144B	NBP2-34588R	NovusBio
UEA I	Dylight 649	N/A	DL-1068	Vector
CD31	APC	WM59	303116	Biolegend
VCAM-1	Dylight 550	6G9	NBP1-47491R	NovusBio
CD62E	unconjugated	polyclonal	PA5-96091	Thermo
Granzyme B	unconjugated	D6E9W	46890S	Cell Signaling
CD3	unconjugated	CD3-12	ab11089	Abcam
CD4	unconjugated	EPR6855	ab133616	Abcam
CD31	unconjugated	JC70A,	GA-610612, CMC131	Dako/Agilent, Biocare Medical
CD68	unconjugated	KP-1	MS397	Thermo Scientific
CD45RO	unconjugated	UCHL-1	CM006	Biocare Medical
CD4	unconjugated	BLR167J	MA5-44519	Invitrogen
CD8	biotin	4SM 15	15-0808-82	Invitrogen
CD11c	unconjugated	5D11	ACI-3122	Biocare Medical
HLA B	unconjugated	polyclonal	PA5-35345	Thermo Scientfic
CD56	unconjugated	JF1021	NBP266968	NovusBio

Supplementary Table 2. Assessed parameters of control groups

	HSC+PBMC	WT EC skin	HSC+WT-EC skin	HSC+KO-EC skin	WT-EC skin+PBMC
circulating hCD45	medium	no	low	low	low
circulating CD3	majority	no	minimal	minimal	majority
circulating CD33	low	no	majority	majority	no
skin graft ly infiltration	N/A	no	no	no	no
skin graft rejection	N/A	no	no	no	no
EC present in skin grafts	N/A	yes	yes	yes	yes
spleen ly infiltration	medium	no	no	no	low-medium
